# Ethyl 3-(4-meth­oxy­phen­yl)-2-phenyl-3-(4-phenyl-1,2,3-selenadiazol-5-yl)propano­ate

**DOI:** 10.1107/S1600536812028322

**Published:** 2012-07-07

**Authors:** P. Sugumar, S. Sankari, P. Manisankar, M. N. Ponnuswamy

**Affiliations:** a Centre of Advanced Study in Crystallography and Biophysics, University of Madras, Guindy Campus, Chennai 600 025, India; b Department of Chemistry, Sri Sarada College for Women (Autonomous), Fairlands, Salem 636 016, India; c Department of Industrial Chemistry, Alagappa University, Karaikudi 630 003, India

## Abstract

In the title compound, C_26_H_24_N_2_O_3_Se, the selenadiazole ring is planar [maximum deviation = 0.002 (2) Å]. The dihedral angle between the selenadiazole ring and the attached phenyl ring is 49.00 (13)°. The crystal structure is stabilized by inter­molecular C—H⋯N and C—H⋯π inter­actions.

## Related literature

For general background to selenadiazole derivatives, see: El-Bahaie *et al.* (1990 ▶); El-Kashef *et al.* (1986 ▶); Kuroda *et al.* (2001 ▶); Padmavathi *et al.* (2002 ▶); Plano *et al.* (2010 ▶). For hydrogen-bond motifs, see: Bernstein *et al.* (1995 ▶).
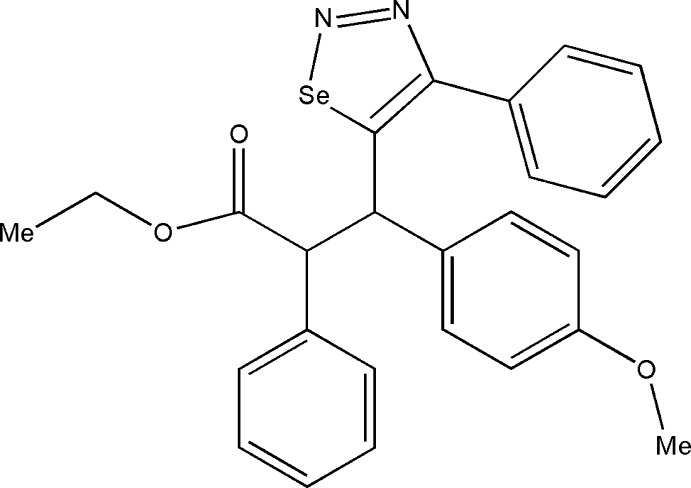


## Experimental

### 

#### Crystal data

C_26_H_24_N_2_O_3_Se
*M* *_r_* = 491.43
Monoclinic, 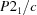

*a* = 11.8187 (4) Å
*b* = 12.8241 (5) Å
*c* = 16.1837 (6) Å
β = 105.280 (2)°
*V* = 2366.16 (15) Å^3^
*Z* = 4
Mo *K*α radiation
μ = 1.62 mm^−1^
*T* = 293 K
0.20 × 0.15 × 0.15 mm

#### Data collection

Bruker SMART APEXII area-detector diffractometer
Absorption correction: multi-scan (*SADABS*; Bruker, 2008 ▶) *T*_min_ = 0.748, *T*_max_ = 0.785
22954 measured reflections
5918 independent reflections
3015 reflections with *I* > 2σ(*I*)
*R*_int_ = 0.046

#### Refinement

*R*[*F* ^2^ > 2σ(*F* ^2^)] = 0.038
*wR*(*F* ^2^) = 0.108
*S* = 0.99
5918 reflections
291 parameters
2 restraints
H-atom parameters constrained
Δρ_max_ = 0.24 e Å^−3^
Δρ_min_ = −0.28 e Å^−3^

### 

Data collection: *APEX2* (Bruker, 2008 ▶); cell refinement: *SAINT* (Bruker, 2008 ▶); data reduction: *SAINT*; program(s) used to solve structure: *SHELXS97* (Sheldrick, 2008 ▶); program(s) used to refine structure: *SHELXL97* (Sheldrick, 2008 ▶); molecular graphics: *ORTEP-3* (Farrugia, 1997 ▶); software used to prepare material for publication: *SHELXL97* and *PLATON* (Spek, 2009 ▶).

## Supplementary Material

Crystal structure: contains datablock(s) global, I. DOI: 10.1107/S1600536812028322/bt5929sup1.cif

Structure factors: contains datablock(s) I. DOI: 10.1107/S1600536812028322/bt5929Isup2.hkl

Supplementary material file. DOI: 10.1107/S1600536812028322/bt5929Isup3.cml

Additional supplementary materials:  crystallographic information; 3D view; checkCIF report

## Figures and Tables

**Table 1:** Hydrogen-bond geometry (Å, °) *Cg*3 and *Cg*4 are the centroids of the C10–C15 and C17–C22 rings, respectively.

*D*—H⋯*A*	*D*—H	H⋯*A*	*D*⋯*A*	*D*—H⋯*A*
C14—H14⋯N1^i^	0.93	2.57	3.420 (3)	152
C12—H12⋯*Cg*4^ii^	0.96	2.81	3.673 (3)	154
C24—H24*A*⋯*Cg*3^iii^	0.96	2.80	3.580 (3)	138

Symmetry code: (i) 
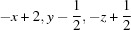
.

**Fig. 1.**
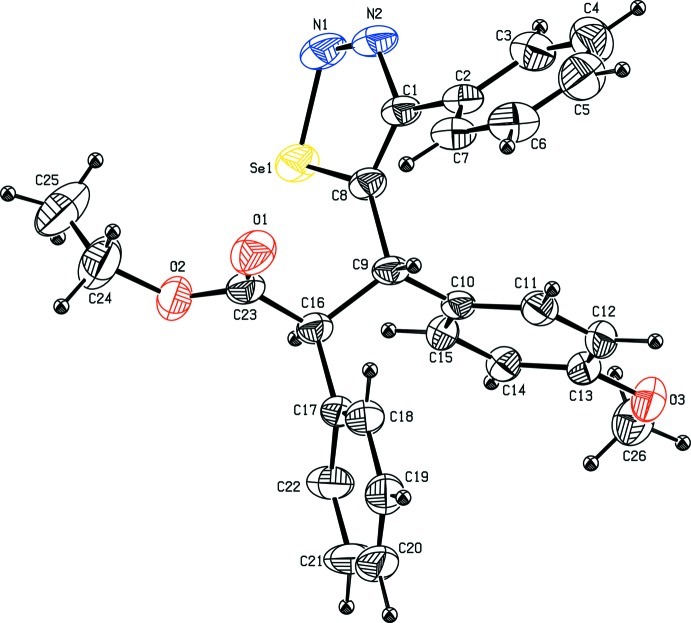
The molecular structure of the title compound, showing the atomic numbering and displacement ellipsoids drawn at 30% probability level.

**Fig. 2.**
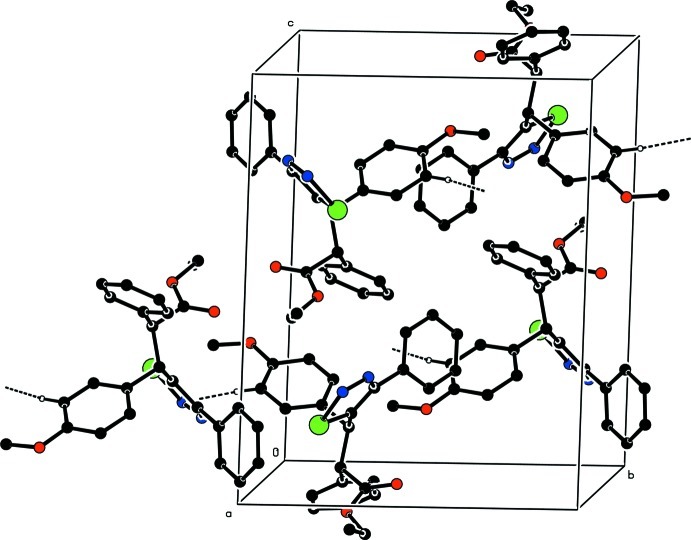
The packing of the molecules viewed down the a axis.

## supplementary crystallographic information

### Comment

Selenium containing compounds, like 1,2,3-selenadiazoles are of increasing interest because of their chemical properties and biological applications such as anti-fungal (Kuroda *et al.*, 2001), anti-bacterial (El-Kashef *et al.*, 1986), anti-microbial (El-Bahaie *et al.*, 1990), anti-cancer (Plano *et al.*, 2010) and insecticidal (Padmavathi *et al.*, 2002) activities. In view of the growing importance of selenium containing compounds, the crystal structure of the title compound has been carried out.

The *ORTEP* plot of the molecule is shown in Fig. 1. The selenadiazol ring is planar(maximum deviation -0.002 (2) Å). The dihedral angle between the selenadiazol ring and the attached phenyl ring(C2—C7) is 49.00 (13)°. The propanoate group assumes an extended conformation which can be seen from the torsion angle (C16—C23—O1—C24) value of 178.4 (2)°. The methoxy group lies in the plane of the phenyl ring (C10—C15) and twisted away with propanate group & phenyl ring (C17—C22) at angles of 8.21 (12)° & 68.11 (12)°, respectively. The packing of the molecules viewed down *a* axis is shown in Fig. 2. The molecules are stabilized by C—H···N and C—H···π types of intermolecular interactions in addition to van der Waals forces.

### Experimental

A mixture of ethyl-3-(4-methoxyphenyl)-5-oxo-2,5-diphenylpentanoate (1 mmol), semicarbazide hydrochloride(2 mmol) and anhydrous sodium acetate (3 mmol) in ethanol (10 ml) was refluxed for 4 hrs. After completion of the reaction as monitored by TLC, the mixture was poured into ice cold water and the resulting semicarbazone was filtered off. Then, a mixtureof semicarbazone (1 mmol) and SeO_2_ (2 mmol) in tetrahydrofuran (10 ml) were refluxed on a water bath for 1 h. The selenium deposited on cooling was removed by filtration, and the filtrate was poured into crushed ice, extracted with dichloromethane, and purified by column chromatography using silica gel (60–120 mesh) with 97:3 petroleum ether: ethyl acetate as eluent to give ethyl 3-(4,5-dihydro-4-phenyl-1,2, 3-selenadiazol-5-yl)-3-(4-methoxyphenyl)-2-phenylpropanoate.

### Refinement

H atoms were positioned geometrically C—H=0.93–0.98 Å) and allowed to ride on their parent atoms,with *U*_iso_(H) = 1.5*U*_eq_(C) for methyl H 1.2*U*_eq_(C) for other H atoms. The *U^ij^* components of atom pairs C19/C20 and C24/C25 in the direction of the bond between them were restrained to be equal within an effective standard deviation of 0.01.

### Crystal data

**Table:**

C_26_H_24_N_2_O_3_Se	*F*(000) = 1008
*M**_r_* = 491.43	*D*_x_ = 1.380 Mg m^−^^3^
Monoclinic, *P*2_1_/*c*	Mo *K*α radiation, λ = 0.71073 Å
Hall symbol: -P 2ybc	Cell parameters from 5918 reflections
*a* = 11.8187 (4) Å	θ = 1.8–28.4°
*b* = 12.8241 (5) Å	µ = 1.62 mm^−^^1^
*c* = 16.1837 (6) Å	*T* = 293 K
β = 105.280 (2)°	Block, white crystalline
*V* = 2366.16 (15) Å^3^	0.20 × 0.15 × 0.15 mm
*Z* = 4	

### Data collection

**Table:**

Bruker SMART APEXII area-detector diffractometer	5918 independent reflections
Radiation source: fine-focus sealed tube	3015 reflections with *I* > 2σ(*I*)
Graphite monochromator	*R*_int_ = 0.046
ω and φ scans	θ_max_ = 28.4°, θ_min_ = 1.8°
Absorption correction: multi-scan (*SADABS*; Bruker, 2008)	*h* = −15→15
*T*_min_ = 0.748, *T*_max_ = 0.785	*k* = −13→17
22954 measured reflections	*l* = −18→21

### Refinement

**Table:**

Refinement on *F*^2^	Primary atom site location: structure-invariant direct methods
Least-squares matrix: full	Secondary atom site location: difference Fourier map
*R*[*F*^2^ > 2σ(*F*^2^)] = 0.038	Hydrogen site location: inferred from neighbouring sites
*wR*(*F*^2^) = 0.108	H-atom parameters constrained
*S* = 0.99	*w* = 1/[σ^2^(*F*_o_^2^) + (0.0454*P*)^2^ + 0.2327*P*] where *P* = (*F*_o_^2^ + 2*F*_c_^2^)/3
5918 reflections	(Δ/σ)_max_ = 0.002
291 parameters	Δρ_max_ = 0.24 e Å^−^^3^
2 restraints	Δρ_min_ = −0.28 e Å^−^^3^

### Special details

**Table:**

Geometry. All e.s.d.'s (except the e.s.d. in the dihedral angle between two l.s. planes) are estimated using the full covariance matrix. The cell e.s.d.'s are taken into account individually in the estimation of e.s.d.'s in distances, angles and torsion angles; correlations between e.s.d.'s in cell parameters are only used when they are defined by crystal symmetry. An approximate (isotropic) treatment of cell e.s.d.'s is used for estimating e.s.d.'s involving l.s. planes.
Refinement. Refinement of *F*^2^ against ALL reflections. The weighted *R*-factor *wR* and goodness of fit *S* are based on *F*^2^, conventional *R*-factors *R* are based on *F*, with *F* set to zero for negative *F*^2^. The threshold expression of *F*^2^ > σ(*F*^2^) is used only for calculating *R*-factors(gt) *etc*. and is not relevant to the choice of reflections for refinement. *R*-factors based on *F*^2^ are statistically about twice as large as those based on *F*, and *R*- factors based on ALL data will be even larger.

### Fractional atomic coordinates and isotropic or equivalent isotropic displacement parameters (Å^2^)

**Table:**

	*x*	*y*	*z*	*U*_iso_*/*U*_eq_	
C1	0.93252 (18)	0.38149 (19)	0.26921 (14)	0.0573 (6)	
C2	0.8634 (2)	0.45552 (18)	0.30523 (15)	0.0600 (6)	
C3	0.8805 (3)	0.4614 (2)	0.39336 (18)	0.0801 (8)	
H3	0.9360	0.4188	0.4292	0.096*	
C4	0.8160 (3)	0.5299 (3)	0.4282 (2)	0.0970 (10)	
H4	0.8284	0.5335	0.4873	0.116*	
C5	0.7334 (3)	0.5929 (3)	0.3758 (3)	0.1004 (10)	
H5	0.6889	0.6381	0.3995	0.120*	
C6	0.7164 (3)	0.5894 (2)	0.2896 (2)	0.0874 (8)	
H6	0.6607	0.6325	0.2544	0.105*	
C7	0.7816 (2)	0.5218 (2)	0.25363 (17)	0.0689 (7)	
H7	0.7706	0.5208	0.1946	0.083*	
C8	0.89231 (18)	0.31086 (19)	0.20497 (14)	0.0552 (6)	
C9	0.76498 (17)	0.28768 (18)	0.15966 (13)	0.0517 (6)	
H9	0.7194	0.3502	0.1642	0.062*	
C10	0.71970 (17)	0.20024 (19)	0.20511 (13)	0.0506 (6)	
C11	0.6465 (2)	0.2215 (2)	0.25734 (16)	0.0621 (6)	
H11	0.6253	0.2902	0.2643	0.075*	
C12	0.6049 (2)	0.1429 (2)	0.29906 (15)	0.0692 (7)	
H12	0.5557	0.1589	0.3337	0.083*	
C13	0.63546 (19)	0.0406 (2)	0.28997 (14)	0.0610 (6)	
C14	0.7102 (2)	0.0182 (2)	0.23980 (14)	0.0602 (6)	
H14	0.7331	−0.0502	0.2341	0.072*	
C15	0.75087 (19)	0.0977 (2)	0.19819 (14)	0.0569 (6)	
H15	0.8010	0.0818	0.1643	0.068*	
C16	0.74743 (19)	0.26514 (18)	0.06321 (14)	0.0532 (6)	
H16	0.7854	0.1984	0.0581	0.064*	
C17	0.6178 (2)	0.25479 (19)	0.01685 (14)	0.0559 (6)	
C18	0.5418 (2)	0.3368 (2)	0.01082 (15)	0.0692 (7)	
H18	0.5694	0.4004	0.0358	0.083*	
C19	0.4241 (2)	0.3260 (3)	−0.03218 (17)	0.0874 (9)	
H19	0.3733	0.3821	−0.0357	0.105*	
C20	0.3833 (3)	0.2340 (4)	−0.06894 (19)	0.1056 (13)	
H20	0.3046	0.2273	−0.0983	0.127*	
C21	0.4568 (3)	0.1517 (3)	−0.0631 (2)	0.1099 (12)	
H21	0.4280	0.0882	−0.0876	0.132*	
C22	0.5750 (2)	0.1614 (2)	−0.02057 (16)	0.0825 (8)	
H22	0.6251	0.1048	−0.0174	0.099*	
C23	0.8048 (2)	0.3473 (2)	0.02199 (15)	0.0636 (6)	
C24	0.9300 (3)	0.3684 (3)	−0.0716 (2)	0.1186 (13)	
H24A	0.8975	0.3616	−0.1330	0.142*	
H24B	0.9233	0.4409	−0.0563	0.142*	
C25	1.0508 (3)	0.3386 (3)	−0.0492 (3)	0.1373 (15)	
H25A	1.0866	0.3579	0.0091	0.206*	
H25B	1.0901	0.3734	−0.0863	0.206*	
H25C	1.0566	0.2645	−0.0555	0.206*	
C26	0.6290 (3)	−0.1371 (3)	0.3307 (2)	0.0979 (10)	
H26A	0.6117	−0.1605	0.2723	0.147*	
H26B	0.5900	−0.1813	0.3624	0.147*	
H26C	0.7121	−0.1399	0.3558	0.147*	
N1	1.11262 (17)	0.3127 (2)	0.27663 (15)	0.0825 (7)	
N2	1.05298 (17)	0.37906 (18)	0.30570 (13)	0.0737 (6)	
O1	0.79677 (18)	0.43897 (17)	0.03213 (13)	0.0920 (6)	
O2	0.86503 (16)	0.30345 (16)	−0.02744 (11)	0.0793 (5)	
O3	0.58939 (16)	−0.03346 (17)	0.33286 (11)	0.0845 (6)	
Se1	1.01611 (2)	0.23384 (2)	0.189123 (19)	0.07749 (14)	

### Atomic displacement parameters (Å^2^)

**Table:**

	*U*^11^	*U*^22^	*U*^33^	*U*^12^	*U*^13^	*U*^23^
C1	0.0423 (12)	0.0620 (15)	0.0588 (14)	−0.0054 (11)	−0.0022 (10)	0.0037 (12)
C2	0.0525 (13)	0.0566 (15)	0.0631 (15)	−0.0116 (12)	0.0011 (11)	−0.0054 (12)
C3	0.087 (2)	0.074 (2)	0.0717 (19)	−0.0048 (16)	0.0061 (15)	0.0003 (15)
C4	0.124 (3)	0.088 (2)	0.084 (2)	−0.012 (2)	0.037 (2)	−0.0146 (19)
C5	0.099 (2)	0.087 (2)	0.123 (3)	0.002 (2)	0.042 (2)	−0.018 (2)
C6	0.0725 (18)	0.071 (2)	0.109 (2)	0.0075 (16)	0.0075 (17)	−0.0109 (18)
C7	0.0575 (14)	0.0651 (17)	0.0728 (16)	−0.0058 (13)	−0.0024 (12)	−0.0041 (14)
C8	0.0386 (11)	0.0635 (15)	0.0582 (13)	0.0012 (11)	0.0032 (10)	0.0024 (12)
C9	0.0339 (10)	0.0581 (15)	0.0572 (13)	0.0005 (10)	0.0018 (9)	−0.0093 (11)
C10	0.0333 (10)	0.0654 (16)	0.0495 (12)	−0.0019 (10)	0.0046 (9)	−0.0100 (11)
C11	0.0460 (12)	0.0768 (17)	0.0615 (15)	0.0070 (13)	0.0105 (11)	−0.0119 (14)
C12	0.0502 (13)	0.103 (2)	0.0595 (15)	−0.0009 (15)	0.0233 (12)	−0.0128 (15)
C13	0.0465 (13)	0.083 (2)	0.0532 (14)	−0.0084 (13)	0.0119 (11)	−0.0031 (13)
C14	0.0530 (13)	0.0678 (16)	0.0602 (14)	−0.0020 (12)	0.0159 (11)	−0.0057 (13)
C15	0.0463 (12)	0.0696 (17)	0.0577 (14)	−0.0014 (12)	0.0190 (11)	−0.0060 (13)
C16	0.0414 (11)	0.0583 (14)	0.0563 (13)	−0.0021 (11)	0.0065 (10)	−0.0027 (11)
C17	0.0450 (12)	0.0755 (18)	0.0434 (12)	−0.0083 (12)	0.0051 (10)	0.0011 (11)
C18	0.0496 (14)	0.091 (2)	0.0614 (15)	−0.0017 (14)	0.0058 (11)	−0.0004 (14)
C19	0.0488 (15)	0.147 (3)	0.0614 (16)	0.0033 (18)	0.0053 (12)	0.0100 (18)
C20	0.0575 (18)	0.185 (4)	0.0605 (18)	−0.036 (2)	−0.0079 (14)	0.014 (2)
C21	0.091 (2)	0.128 (3)	0.089 (2)	−0.052 (2)	−0.0134 (19)	−0.009 (2)
C22	0.0764 (18)	0.083 (2)	0.0766 (17)	−0.0195 (16)	−0.0004 (15)	−0.0111 (16)
C23	0.0447 (13)	0.079 (2)	0.0600 (15)	−0.0092 (14)	0.0021 (11)	0.0016 (15)
C24	0.080 (2)	0.169 (4)	0.110 (2)	−0.011 (2)	0.0330 (19)	0.049 (2)
C25	0.097 (3)	0.108 (3)	0.229 (5)	−0.029 (2)	0.082 (3)	−0.020 (3)
C26	0.105 (2)	0.098 (3)	0.097 (2)	−0.017 (2)	0.0379 (19)	0.0164 (19)
N1	0.0399 (11)	0.1018 (18)	0.0956 (17)	−0.0025 (12)	0.0000 (11)	0.0053 (14)
N2	0.0448 (11)	0.0841 (16)	0.0790 (14)	−0.0109 (11)	−0.0068 (10)	0.0003 (12)
O1	0.0964 (15)	0.0734 (14)	0.1110 (16)	−0.0179 (12)	0.0357 (12)	0.0019 (12)
O2	0.0643 (11)	0.1052 (14)	0.0741 (11)	−0.0009 (11)	0.0280 (10)	0.0144 (11)
O3	0.0791 (12)	0.1050 (16)	0.0787 (12)	−0.0122 (12)	0.0376 (10)	0.0076 (11)
Se1	0.04128 (15)	0.0978 (3)	0.0883 (2)	0.00942 (14)	0.00818 (13)	−0.00765 (16)

### Geometric parameters (Å, º)

**Table:**

C1—C8	1.366 (3)	C16—C23	1.502 (3)
C1—N2	1.390 (3)	C16—C17	1.524 (3)
C1—C2	1.470 (3)	C16—H16	0.9800
C2—C7	1.388 (3)	C17—C18	1.369 (3)
C2—C3	1.389 (3)	C17—C22	1.377 (3)
C3—C4	1.377 (4)	C18—C19	1.389 (3)
C3—H3	0.9300	C18—H18	0.9300
C4—C5	1.375 (4)	C19—C20	1.351 (5)
C4—H4	0.9300	C19—H19	0.9300
C5—C6	1.357 (4)	C20—C21	1.355 (5)
C5—H5	0.9300	C20—H20	0.9300
C6—C7	1.386 (4)	C21—C22	1.390 (4)
C6—H6	0.9300	C21—H21	0.9300
C7—H7	0.9300	C22—H22	0.9300
C8—C9	1.520 (3)	C23—O1	1.194 (3)
C8—Se1	1.838 (2)	C23—O2	1.328 (3)
C9—C10	1.514 (3)	C24—C25	1.429 (4)
C9—C16	1.547 (3)	C24—O2	1.444 (3)
C9—H9	0.9800	C24—H24A	0.9700
C10—C15	1.378 (3)	C24—H24B	0.9700
C10—C11	1.386 (3)	C25—H25A	0.9600
C11—C12	1.375 (3)	C25—H25B	0.9600
C11—H11	0.9300	C25—H25C	0.9600
C12—C13	1.380 (4)	C26—O3	1.412 (4)
C12—H12	0.9300	C26—H26A	0.9600
C13—O3	1.371 (3)	C26—H26B	0.9600
C13—C14	1.378 (3)	C26—H26C	0.9600
C14—C15	1.377 (3)	N1—N2	1.272 (3)
C14—H14	0.9300	N1—Se1	1.865 (2)
C15—H15	0.9300		
			
C8—C1—N2	114.9 (2)	C23—C16—C9	111.16 (19)
C8—C1—C2	127.72 (19)	C17—C16—C9	111.29 (18)
N2—C1—C2	117.4 (2)	C23—C16—H16	107.8
C7—C2—C3	118.3 (3)	C17—C16—H16	107.8
C7—C2—C1	121.9 (2)	C9—C16—H16	107.8
C3—C2—C1	119.8 (2)	C18—C17—C22	118.6 (2)
C4—C3—C2	120.6 (3)	C18—C17—C16	121.6 (2)
C4—C3—H3	119.7	C22—C17—C16	119.8 (2)
C2—C3—H3	119.7	C17—C18—C19	120.7 (3)
C5—C4—C3	120.1 (3)	C17—C18—H18	119.7
C5—C4—H4	119.9	C19—C18—H18	119.7
C3—C4—H4	119.9	C20—C19—C18	120.1 (3)
C6—C5—C4	120.2 (3)	C20—C19—H19	119.9
C6—C5—H5	119.9	C18—C19—H19	119.9
C4—C5—H5	119.9	C19—C20—C21	120.1 (3)
C5—C6—C7	120.3 (3)	C19—C20—H20	120.0
C5—C6—H6	119.9	C21—C20—H20	120.0
C7—C6—H6	119.9	C20—C21—C22	120.5 (3)
C6—C7—C2	120.4 (3)	C20—C21—H21	119.8
C6—C7—H7	119.8	C22—C21—H21	119.8
C2—C7—H7	119.8	C17—C22—C21	120.0 (3)
C1—C8—C9	126.8 (2)	C17—C22—H22	120.0
C1—C8—Se1	109.41 (16)	C21—C22—H22	120.0
C9—C8—Se1	123.43 (17)	O1—C23—O2	125.1 (2)
C10—C9—C8	110.02 (17)	O1—C23—C16	124.6 (3)
C10—C9—C16	112.41 (17)	O2—C23—C16	110.3 (2)
C8—C9—C16	112.08 (18)	C25—C24—O2	110.2 (3)
C10—C9—H9	107.4	C25—C24—H24A	109.6
C8—C9—H9	107.4	O2—C24—H24A	109.6
C16—C9—H9	107.4	C25—C24—H24B	109.6
C15—C10—C11	117.4 (2)	O2—C24—H24B	109.6
C15—C10—C9	122.1 (2)	H24A—C24—H24B	108.1
C11—C10—C9	120.5 (2)	C24—C25—H25A	109.5
C12—C11—C10	121.1 (2)	C24—C25—H25B	109.5
C12—C11—H11	119.4	H25A—C25—H25B	109.5
C10—C11—H11	119.4	C24—C25—H25C	109.5
C11—C12—C13	120.5 (2)	H25A—C25—H25C	109.5
C11—C12—H12	119.8	H25B—C25—H25C	109.5
C13—C12—H12	119.8	O3—C26—H26A	109.5
O3—C13—C14	123.8 (3)	O3—C26—H26B	109.5
O3—C13—C12	117.1 (2)	H26A—C26—H26B	109.5
C14—C13—C12	119.2 (2)	O3—C26—H26C	109.5
C15—C14—C13	119.6 (2)	H26A—C26—H26C	109.5
C15—C14—H14	120.2	H26B—C26—H26C	109.5
C13—C14—H14	120.2	N2—N1—Se1	110.86 (15)
C14—C15—C10	122.1 (2)	N1—N2—C1	117.6 (2)
C14—C15—H15	118.9	C23—O2—C24	119.5 (3)
C10—C15—H15	118.9	C13—O3—C26	117.4 (2)
C23—C16—C17	110.84 (18)	C8—Se1—N1	87.29 (10)
			
C8—C1—C2—C7	−51.0 (4)	C9—C10—C15—C14	179.68 (19)
N2—C1—C2—C7	131.4 (2)	C10—C9—C16—C23	−173.75 (19)
C8—C1—C2—C3	129.9 (3)	C8—C9—C16—C23	−49.2 (3)
N2—C1—C2—C3	−47.6 (3)	C10—C9—C16—C17	62.2 (2)
C7—C2—C3—C4	1.3 (4)	C8—C9—C16—C17	−173.29 (19)
C1—C2—C3—C4	−179.6 (2)	C23—C16—C17—C18	−60.5 (3)
C2—C3—C4—C5	0.3 (5)	C9—C16—C17—C18	63.8 (3)
C3—C4—C5—C6	−1.2 (5)	C23—C16—C17—C22	119.2 (3)
C4—C5—C6—C7	0.4 (5)	C9—C16—C17—C22	−116.6 (2)
C5—C6—C7—C2	1.2 (4)	C22—C17—C18—C19	0.0 (4)
C3—C2—C7—C6	−2.1 (4)	C16—C17—C18—C19	179.6 (2)
C1—C2—C7—C6	178.9 (2)	C17—C18—C19—C20	−0.3 (4)
N2—C1—C8—C9	173.3 (2)	C18—C19—C20—C21	0.8 (5)
C2—C1—C8—C9	−4.3 (4)	C19—C20—C21—C22	−1.1 (5)
N2—C1—C8—Se1	0.4 (3)	C18—C17—C22—C21	−0.3 (4)
C2—C1—C8—Se1	−177.27 (19)	C16—C17—C22—C21	−179.9 (3)
C1—C8—C9—C10	−91.0 (3)	C20—C21—C22—C17	0.8 (5)
Se1—C8—C9—C10	81.0 (2)	C17—C16—C23—O1	78.4 (3)
C1—C8—C9—C16	143.1 (2)	C9—C16—C23—O1	−46.0 (3)
Se1—C8—C9—C16	−44.8 (3)	C17—C16—C23—O2	−101.3 (2)
C8—C9—C10—C15	−74.9 (3)	C9—C16—C23—O2	134.35 (19)
C16—C9—C10—C15	50.8 (3)	Se1—N1—N2—C1	−0.1 (3)
C8—C9—C10—C11	103.6 (2)	C8—C1—N2—N1	−0.2 (3)
C16—C9—C10—C11	−130.7 (2)	C2—C1—N2—N1	177.7 (2)
C15—C10—C11—C12	−1.4 (3)	O1—C23—O2—C24	2.0 (4)
C9—C10—C11—C12	−179.9 (2)	C16—C23—O2—C24	−178.4 (2)
C10—C11—C12—C13	0.2 (4)	C25—C24—O2—C23	123.3 (3)
C11—C12—C13—O3	−179.3 (2)	C14—C13—O3—C26	5.4 (3)
C11—C12—C13—C14	1.2 (3)	C12—C13—O3—C26	−174.0 (2)
O3—C13—C14—C15	179.1 (2)	C1—C8—Se1—N1	−0.32 (18)
C12—C13—C14—C15	−1.5 (3)	C9—C8—Se1—N1	−173.5 (2)
C13—C14—C15—C10	0.3 (3)	N2—N1—Se1—C8	0.2 (2)
C11—C10—C15—C14	1.1 (3)		

### Hydrogen-bond geometry (Å, º)

**Table:**

*D*—H···*A*	*D*—H	H···*A*	*D*···*A*	*D*—H···*A*
C14—H14···N1^i^	0.93	2.57	3.420 (3)	152
C12—H12···*Cg*4^ii^	0.96	2.81	3.673 (3)	154
C24—H24*A*···*Cg*3^iii^	0.96	2.80	3.580 (3)	138

Symmetry codes: (i) −*x*+2, *y*−1/2, −*z*+1/2; (ii) *x*, −*y*+1/2, *z*+1/2; (iii) *x*, −*y*+1/2, *z*−1/2.
